# The hyper-systemizing hypothesis: how the tendency to systemize influences conspiracy beliefs and belief inflexibility in clinical and general populations

**DOI:** 10.1007/s10339-025-01326-0

**Published:** 2026-01-14

**Authors:** Neophytos Georgiou, Paul Delfabbro, Ryan P. Balzan, Nathan Caruana, Robyn Young

**Affiliations:** 1 College of Education, Psychology and Social Work, Flinders University, GPO Box 2100, Adelaide, SA 5001 Australia; 2 School of Psychology, Faculty of Health and Medical Sciences, University of Adelaide, Adelaide, Australia

**Keywords:** Conspiracy beliefs, Systemising, Thinking styles, Scientific reasoning, Bias against confirmatory evidence, Autism

## Abstract

Endorsing conspiracy beliefs may relate to fixated interests and less flexible thinking—patterns often observed in individuals with higher autistic traits and autism spectrum disorder. This study examines whether systemizing tendencies, measured by the Systemizing Quotient, contribute to conspiracy belief formation. Study 1, in a general population sample, identified two latent profiles with high autistic traits: one with strong systemizing, better scientific reasoning, and greater conspiracy endorsement; another with lower systemizing, weaker scientific reasoning, but similarly high conspiracy beliefs. These contrasting profiles suggest different cognitive pathways to conspiracy beliefs. Study 2, in an autistic sample, found that systemizing moderated the relationship between autistic traits and conspiracy beliefs, with an interaction between belief inflexibility and systemizing. Overall, these findings suggest systemizing shapes distinct cognitive profiles in individuals with high autistic traits, influencing scientific reasoning and conspiracy endorsement. Systemizing emerged as a key predictor, supporting the hyper-systemizing hypothesis as a distinct pathway to conspiracy beliefs.

**Supplementary Information:**

The online version contains supplementary material available at 10.1007/s10339-025-01326-0.

## Introduction

Conspiracy theories (CTs) often provide straightforward explanations for complex world events (De Coninck et al. 2021; Douglas et al. 2017). For example, many conspiracy theories about global powers are rooted in the belief that corruption or malicious intent drives major world events—a narrative that can be applied to any number of actors and contexts (Douglas et al. 2023; Nera et al. 2024; Uscinski et al. 2022). Such views are often seen to simplify how the world operates, and this often manifests in the emergence of *monolithic belief* structures in which multiple conspiracy theories converge into a single, overarching narrative (Jolley et al. 2022; Zeng et al. 2022). The prevalence of CTs, particularly those which have implications for public health and the respect for democratic institutions, has led to interest in what individual differences lead certain individuals to be more prone to these beliefs than others (Jolley et al. 2022).

Cognitive research in this area has explored how various thinking styles and traits influence susceptibility to conspiracy theory beliefs (Denovan et al. 2018; Goreis and Voracek 2019). Specifically, it highlights how cognitive patterns, such as biased information processing or insufficient critical assessment of CT content, can lead to the formation of poorly evaluated beliefs (Čavojová et al. 2020; Georgiou et al. 2019, 2020, 2021a, b, c, d; Mikušková and Čavojová 2020; Pennycook and Rand 2020; van Prooijen and Milošević-Đorđević 2024). The shared view in CT research suggests that individuals who engage less in analytical thinking (i.e., systematically seeking out and critically evaluating information) and have lower levels of education are less likely to challenge CT beliefs effectively (Epstein and Norris 2011; Lantian et al. 2021; Stahl and van Prooijen 2018).

However, recent research by Georgiou et al. (2021c) presents a paradox: individuals with higher autistic traits, despite a preference for analytical thinking, were more likely to endorse conspiracy theories and show a bias against counterevidence that would debunk CTs. This suggests that a preference for systematic and consistent thinking does not always involve sufficient critique of one’s beliefs and may leave people vulnerable to cognitive biases (Čavojová et al. 2020a, b; Gjoneska et al. 2021; Georgiou et al. 2021c; Pennycook et al. 2020; Pennycook and Rand 2021; Pytlik et al. 2020; Roberts and Risen et al. 2022; Rutjen et al. 2022). In fact, this preliminary research suggests that a preference for more systematic thinking could serve as a pathway to conspiracy beliefs and a biased interpretation of conspiracy content, with research on autistic traits offering a novel example of how this occurs. Evidence indicates that traits such as a preference for sameness (i.e., a need for consistency and predictability) and a tendency to focus on specific details rather than broader, more abstract concepts (often referred to as "big picture" thinking) may underlie this relationship (Georgiou et al. 2021c; 2022; 2024). Furthermore, Georgiou et al. (2024) demonstrated that, within the general population, the relationship between Autism Quotient scores and higher endorsement of conspiracy theories in a simulated social media environment was mediated by a preference for a more analytical, yet biased thinking style, as revealed through path analysis.

While the Autism Quotient has been used in misinformation research, a limitation is that it captures a range of behaviour which may extend beyond the specific qualities thought to increase susceptibility for CTs. Reported effects have generally been small and sometimes mediated by cognitive factors (Georgiou et al. 2022, 2023). Indeed, some research suggests that separating individuals based on an autism diagnosis alone does not increase the likelihood of endorsing conspiracy theories (Roels et al. 2024). Consequently, this raises the question of whether a more targeted approach, using a measure specifically designed to assess systemizing tendencies (ST), might provide deeper insight into the relationship between systematic thinking and conspiracy beliefs in both autistic and general populations. STs refer to attempts to find meaning and the logic behind every day phenomena and to organise information into systems (Brosnan et al. 2023; Park et al. 2023; Sindermann 2019,, Williams et al. 2014). Although such thinking may be common to scientific approaches to research, it could also be a cognitive tendency that makes certain individual more prone to conspiracy narratives. Individuals with strong ST may find it more difficult to see events as unconnected, random, or illogical, and therefore make greater attempts to find a way to explain why certain events occur (Baron-Cohen 2008; Caldwell-Harris and Jordan 2014). Conceptually, we refer to this as the ‘hyper-systemising’ hypothesis for conspiracy theory beliefs.

### The present study

The current study seeks to address the limitations of prior research by investigating whether the Systemizing Quotient (SQ) provides a more precise measure of traits associated with conspiracy beliefs and a bias toward counterevidence, and whether this relationship is particularly relevant within autistic populations*. Study 1* employed a Latent Profile Analysis (LPA) to compare profiles of individuals from a general sample, evaluating whether the SQ more effectively captures traits linked to conspiracy beliefs across different groups. Following, *Study 2* assessed the SQ's predictive value within a clinical sample of individuals diagnosed with autism, examining whether it outperforms broader measures, such as the Autism Quotient (AQ), in its association with conspiracy beliefs. Additionally, Study 2 will explore whether systemizing tendencies (ST) explain the relationship between AQ and conspiracy theory (CT) beliefs. Together, this approach will test the validity of the 'hyper-systemizing hypothesis,' which posits that a preference for systemizing acts as a distinct pathway to conspiracy beliefs and susceptibility to biases against contrary evidence that would debunk conspiracy theories (e.g., the Bias Against Disconfirmatory Evidence task; Georgiou et al. 2023, 2025). Specifically, we hypothesise that individuals with systemising tendencies in both autistic and general populations, will show a higher level of conspiracy theory endorsement as conspiracy theories provide the sense of predictability and control that align with a more systematic belief system.

## Study 1

### Method

Study 1 involved 412 participants (Male = 220, Female = 192). Participants were recruited from a range of countries, with the majority residing within the United States of America (23%), the United Kingdom (18%), or Continental Europe (16%). Other demographic results including, level of education and prior mental health history can be found in Table 1.

**Table 1:** Demographic characteristics of study 1 (*N* = 412)

	*N* (%)
*Gender*	
Male	220 (53.5)
Female	192 (46.5)
Not stated	0
*Age*	
18–24	134 (32.5)
25–34	113 (27.5)
35–44	97 (23.5)
45–54	55 (13.5)
55–64	13 (3.0)
65+	0
*Country*	
Oceania	32 (4.5)
United Kingdom	192 (27.4)
United States of America	219 (31.2)
Canada	6 (0.9)
South Africa	34 (4.8)
Other continental Europe	146 (20.8)
Rest of the world (nations with, N < 5)	71 (10.1)
*Education*	
University degree	152 (37.0)
Some college	130 (31.5)
High school only	99 (24.0)
Less than high school	31 (7.5)
*Clinical history*	
Received prior diagnosis	58 (14.0)
ASD only	12 (3.0)
No formal diagnosis	342 (83.0)

### Sampling procedure

The online research participation website *Cloud Research* was used to recruit participants, and the study was advertised as being about ‘people’s way of interpreting real-world events’. There was a small monetary fee (around $7US). Participants were provided and completed written consent before commencing the study.

### Measures

#### Autism-spectrum quotient: short form (AQ-10)

The AQ-10 is a shortened, 10-item version of the original 10-item Autism-Spectrum Quotient (Baron-Cohen 2008), developed by Hoekstra et al. (2011). It measures the extent to which adults with average intelligence exhibit traits associated with the autism spectrum. Participants responded on a Likert scale from 1 (definitely disagree) to 4 (definitely agree), following the scoring recommendations of Stevenson and Hart (2017) for predominantly neurotypical populations (e.g., college students). This scale has also been validated for use with the general population in studies on autistic traits and conspiracy theory beliefs (Georgiou et al. 2021a). In this sample, the AQ-10 demonstrated good internal reliability (Cronbach’s alpha = 0.86).

#### Systemizing quotient-revised (SQ-R)

The SQ-R is a 10-item shortened version of the original 75-item Systemizing Quotient (Wheelwright et al. 2006), developed by Greenberg et al. (2018) to assess systemizing cognitive styles. Systemizing refers to the drive to analyse or construct systems of thought or behaviour, a tendency especially common among individuals with Autism Spectrum Disorder. Participants rated each item on a Likert scale ranging from 1 (strongly disagree) to 4 (strongly agree). Higher scores indicate a stronger attention to detail, a preference for discerning causal patterns, and a tendency toward organized behaviour. Baron-Cohen (2008) notes that systemizing functions to predict lawful events and provide consistent patterns for understanding life events (Cronbach’s alpha = 0.88).

#### Bias against disconfirmatory evidence (BADE) task

Biased evidence integration was assessed using the BADE task developed by Woodward et al. (2006). In this study, a single set of 6 text-based scenarios was presented to evaluate participants' ability to integrate evidence and adjust their beliefs. Each scenario includes four interpretations rated for plausibility after each of three successively informative statements. Two of the interpretations are “lures”, plausible initially but becoming unlikely as more information is provided; one is the “true” interpretation, which may seem implausible at first but is confirmed as most likely by the final statement; and one is an “absurd” interpretation that remains implausible throughout.

For example, the first statement ‘Jenny can’t fall asleep’, may be interpreted in several ways (‘Jenny is nervous about her exam the next day’ or ‘Jenny is worried about her ill mother’). However, as more statements are presented (‘Jenny can’t wait until it is finally morning’), it becomes increasingly apparent which interpretations are lures, and which is the true interpretation (i.e., ‘Jenny is excited about Christmas morning’). The *change* in plausibility ratings for the lure and true interpretations across the three statements gauges how participants respond to the disconfirmatory evidence. Those diagnosed with ASD or those with higher levels of autistic traits (non-clinical) have shown an association with the failure to *downrate* lure interpretations (bias against disconfirmatory bias or BADE) and *uprate* true interpretations appropriately over time (bias against confirmatory evidence or BACE). A final metric was an evidence integration (EI) score based on an algorithm outlined in Sanford et al. (2014), and calculation validated within Georgiou et al. (2025b, under review) were applied within the present study.

#### Scientific reasoning ability (scientific reasoning scale; SRS)

Scientific reasoning skills were assessed using the Scientific Reasoning Scale (SRS) developed by Drummond and Fischhoff (2017), as used in studies by Georgiou et al. (2021c, 2023). The SRS comprises 11 scenarios that measure participants' understanding of core scientific concepts, such as confounding variables, control group effects, and random assignment. For example, one scenario on confounding variables describes a situation in which participants complete a jigsaw puzzle in either a cold room with a loud radio or a warm room with no radio, with subjects solving the puzzle faster in the warm room. Participants then assess statements such as “The scientist cannot tell if the radio caused subjects to solve the puzzle more slowly,” indicating whether the statement is true, false, or selecting “I don’t know” to minimize random guessing. The total SRS score, ranging from 0 to 11, is the sum of correct responses, with Cronbach’s alpha of 0.82 in this sample.

#### Conspiracy theory beliefs (generic conspiracy belief scale; GCBS)

The GCBS is a 15-item self-report scale developed by Brotherton et al. (2013) to assess the tendency to interpret events through a conspiratorial perspective, without focusing on specific conspiracy theories (Swami et al. 2017). An example item is “Some of the people thought to be responsible for acts of terrorism were actually set up by those responsible.” Items are rated from 1 (definitely not true) to 5 (definitely true), yielding a total score between 15 and 75. In this sample, the GCBS demonstrated strong internal consistency (Cronbach’s alpha = 0.85).

### Study design

Demographic information and other measures, (including screening for prior clinical diagnosis) and measures of both scientific reasoning performance and BADE task performance were completed online. The study was approved by the Research Ethics Committee of **anonymised**.

### Latent profile analysis

For the purposes of exploratory LPA, in accordance with the recommendations of previous research (i.e. Deleuze et al. 2015; Hussain et al. 2015; Denovan et al. 2018), psychometric measures using Likert scales were recoded. On measures of autistic traits (AQ scores), systemising tendencies (SQ scores) and conspiracy endorsement (GCBS scores), scores indicating disagreement or uncertainty (i.e., scores of 0–3) were recorded as “0”, and scores indicating agreement 4–5 were coded as “1”. Evidence integration scores were recoded to a range of − 10 to 10 as per previous research applying class analysis to the performance task. Scientific reasoning performance was not recoded.

The optimal number of latent classes was determined by considering a range of indices; the Akaike Information Criterion (AIC; Akaike 1987), the Bayesian Information Criterion (BIC; Schwarz 1978) the sample-size adjusted BIC (ssaBIC; Sclove 1987), the Lo-Mendell-Rubin-adjusted likelihood radio test (LMR-A-LRT; Lo et al. 2001), and a standardized measure of entropy (Ramaswamy et al. 1993). For AIC, BIC, and ssaBIC scores, smaller value indicates a better fit of the model. The LMR-A-LRT score does not rely on chi-square distribution for the difference in model likelihood values and normally occur alongside an associated *p*-value. Progressive class solutions are computed until an LMR-A-LRT value is found that is non-significant, which indicates the model cannot be improved for fit. Lastly, entropy ranges from 0 to 1, with higher values suggesting a better classification of participants. According to Ramaswamy et al. (1993), an entropy value of above 0.80 reflects a sound separation of identified classes in relation to the data. Once LPA exploratory analysis has been conducted, Latent Class membership acted as a group variable for assessing whether differences existed on scientific reasoning and BADE scores.

## Results

A summary of the demographic and descriptive statistics for the measures used in this study, and performance on both the scientific reasoning task and each outcome as a result of the BADE task are summarised in Tables 1 and 2. From this sample, 31 participants scored above the AQ cut-off for potentially being within a clinical range of autistic traits. Of the 31 participants, only nine participants had reported receiving a formal diagnosis for Autism Spectrum Disorder. This is unsurprising considering the documented incongruence between AQ-cut off scores and confirmed diagnosis within online research (Ashwood et al. 2016).

**Table 2:** Descriptive statistics for psychometric measures

	*M* (*SD*)	Range of participant scores (range)
AQ-10	21.45 (3.54)	12–38 (10–40)
SQ-10	25.99 (4.04)	17–38 (10–40)
*BADE task*		
Liberal acceptance	10.88 (12.44)	0–65 (0–100)
BADE	45.78 (18.99)	− 18–95 (− 100–100)
BACE	52. 77 (21.33)	0–100 (− 100–100)
SRS	4.35 (2.81)	2–11 (0–11)
GCBS	39.77 (17.00)	15–75 (15–75)

*AQ = 10* Autism quotient-10, *SQ-10* Systemizing quotient-10, *BADE task* Bias against disconfirmatory evidence task, *SRS* Scientific reasoning scale, *GCBS* General conspiracy belief scale

### Latent profile analysis

Prior to conducting the latent profile analysis (LPA), potential covariates of conspiracy theory (CT) beliefs, including level of education, age, gender, and prior clinical diagnosis, were examined to assess their influence on the parameters of interest (e.g., AQ, SQ, SRS, GCBS scores). There was no gender difference in GCBS, t(410) = 0.77, p = 0.44, d = 0.08. GCBS also did not differ across education levels, F(3, 408) = 1.03, p = 0.38, η^2^ = 0.008, or across age groups, F(4, 407) = 0.94, p = 0.44, η^2^ = 0.009. Consequently, gender, age, and education were not retained as covariates in the LPA.

Initial model comparisons began with 1-class and 2-class solutions. Model fit indices, including AIC, BIC, and ssaBIC, indicated that the 2-class model provided a superior fit. Additionally, the Lo-Mendell-Rubin adjusted likelihood ratio test (LMR-A-LRT) for the 2-class model demonstrated a significant improvement over the 1-class model (see Table 3). Subsequent comparison between 2-class and 3-class solutions favoured the 3-class model, as evidenced by lower AIC, BIC, and ssaBIC values, higher entropy scores (0.77 compared to 0.71), and a significant LMR-A-LRT p-value. Further examination of a 4-class solution suggested it provided an even better fit, with reduced AIC, BIC, and ssaBIC values, increased entropy scores (0.82 compared to 0.77), and a significant LMR-A-LRT p-value. However, exploration of a 5-class solution did not yield significant improvement over the 4-class model. When these tests fail to reach significance, the more parsimonious model is typically preferred to avoid overextraction and ensure replicability (Berlin et al. 2014; Masyn 2013; Nylund et al. 2007). Consequently, the 4-class solution was selected as the final model, as it represented the optimal balance of fit indices and interpretability.

**Table 3:** Mean score (standard deviations) of all psychometric measures as a function of latent class in study 1

Model	AIC	BIC	ssaBIC	LMR-A	LMR-A (*p*-value)	Entropy
1-class	34,288.72	34,550.30	34,472.05			
2-class	30,045.11	30,120.62	30,203.20	780.22	<0.001	0.71
3-class	28,461.42	28,731.11	28,798.44	610.33	<0.001	0.77
4-class	25,321.77	25,861.03	25,799.31	255.10	<0.001	0.82
5-class	24,888.52	25,130.27	25,633.82	103.22	0.091	0.83

*AIC* Akaike information criterion, *BIC* Bayesian information criterion, *ssaBIC* Sample-size adjustment BIC, *LMR-A* Lo-Mendell-Rubin-adjusted likelihood ratio test

As shown in Table 4, a 4-class solution represented the model most likely to be of best fit. In this model, *Class 1* (37.6% of the sample) demonstrated low scores on autistic traits, systemizing tendencies, and conspiracy beliefs, along with the highest levels of scientific reasoning skills and best performance on the BADE task (i.e., lowest evidence integration scores). *Class 1* can be considered the group with the strongest reasoning abilities and the least likely to endorse conspiracy theories. *Class 2* (29.6% of the sample) also had low scores on autistic traits and systemizing tendencies but showed slightly lower scientific reasoning and BADE performance. Although *Class 2* exhibited less adept reasoning skills, they did not show ‘clinical traits’ (i.e., did not score above the cut-off range on the AQ or SQ for likely clinical presentation) or a heightened tendency to endorse conspiracy beliefs. *Class 3*, representing 14.3% of the sample, was most distinct in exhibiting the highest systemizing tendencies, intermediate levels of autistic traits and conspiracy beliefs, and lower evidence integration ability on the BADE task. However, their scientific reasoning performance was similar to that of *Class 2*. *Class 4* (18.4% of the sample) exhibited the highest levels of autistic traits and conspiracy beliefs, coupled with the lowest scientific reasoning scores and poorest BADE task performance (i.e., the lowest ability to integrate evidence). Both *Class 3* and *Class 4* may represent ‘clinical’ levels of autistic traits, but key differences in systemizing tendencies, reasoning skills, and BADE performance may distinguish the two groups.

**Table 4:** Mean score (standard deviations) of all psychometric measures as a function of latent class

	Class 1 (*n* = 155)	Class 2 (*n* = 122)	Class 3 (*n* = 59)	Class 4 (*n* = 76)
AQ-10	2.01 (1.03)	2.44 (0.96)	5.21 (0.78)	7.66 (1.58)
SQ-10	1.30 (0.84)	3.12 (1.04)	8.11 (2.12)	5.20 (1.83)
SRS	7.55 (1.12)	6.58 (1.44)	6.31 (1.85)	3.66 (2.10)
BADE	− 1.52 (1.39)	0.51 (2.06)	1.62 (1.76)	2.92 (1.64)
GCBS	3.86 (2.23)	4.20 (2.50)	6.38 (1.17)	7.56 (2.83)

Pattern of mean scores as a function of latent class models across the Autism quotient-10, AQ-10; Systemising quotient-10, SQ; *SRS* Scientific reasoning scale, Evidence integration score on the bias against disconfirmatory evidence task, BADE; General conspiracy belief scale, GCBS

As shown in Table 5, post-hoc pairwise comparisons with Bonferroni correction were used to assess AQ, SQ, SRS, conspiracy theory beliefs, and BADE performance as a function of class membership. In terms of conspiracy theory endorsement (via GCBS scores), Bonferroni correction revealed that Class 3 and Class 4, which likely represent individuals with clinical levels of autistic traits, had significantly higher conspiracy belief scores compared to Class 1 and Class 2. The difference in scores between Class 4 and Class 1 (i.e., those with the highest level of scientific reasoning) highlights the expected pattern from the existing literature: the more adept individuals are at evaluating conspiracy content, the less likely they are to endorse CTs. However, the results here suggest that Class 3 has demonstrated an exception to this argument with both higher SRS and higher CT endorsement.

**Table 5:** Pairwise comparisons (mean differences) performance as a function of class membership

Class contrast	GCBS mean diff. (sig.)	SRS mean diff. (sig.)	BADE mean diff. (sig.)	AQ-10 mean diff. (sig.)	SQ-10 mean diff. (sig.)
Class 1 vs. class 2	0.34 (0.382)	− 0.97 (0.031)	− 2.03 (0.042)	− 0.43 (0.061)	− 1.82 (0.014)
Class 1 vs. class 3	2.52 (0.001)	− 1.24 (0.055)	− 3.14 (0.020)	− 3.20 (0.001)	− 6.81 (0.001)
Class 1 vs. class 4	3.70 (0.001)	− 3.89 (0.001)	− 4.44 (0.001)	− 5.65 (0.001)	− 3.90 (0.001)
Class 2 vs. class 3	2.18 (0.001)	− 0.27 (0.531)	− 1.11 (0.089)	− 2.77 (0.001)	− 4.99 (0.001)
Class 2 vs. class 4	3.36 (0.001)	− 2.92 (0.001)	− 2.41 (0.026)	− 5.22 (0.001)	− 2.08 (0.017)
Class 3 vs. class 4	1.02 (0.240)	− 2.65 (0.002)	− 1.30 (0.077)	− 2.45 (0.001)	2.91 (0.046)

*GCBS* General conspiracy belief scale, *SRS* Scientific reasoning scale, *BADE* Bias against disconfirmatory evidence task, *AQ-10* Autism quotient-10, *SQ-10* Systemizing quotient-10

## Study 1: discussion

The results of Study 1 demonstrate that among latent profiles characterized by likely 'clinical' levels of autistic traits, there is variability in systemizing tendencies, scientific reasoning, and susceptibility to cognitive bias. Despite this variability, both profiles with elevated autistic traits were more likely to endorse conspiracy theories compared to other latent profiles in the final model of fit. Specifically, Class 3 exhibited significantly higher engagement in systemizing tendencies and stronger scientific reasoning skills compared to Class 4, that did not statistically differ to Class 2 or Class 1. These findings suggest that individuals with elevated autistic traits and a stronger inclination toward systemizing tendencies may simultaneously perform well on scientific reasoning tasks and endorse conspiracy theories. Compared to previous latent profile analyses (e.g., Georgiou et al. 2022), this study further supports the notion that there is some diversity in the performance of people with autistic traits, and as such different cognitive pathways may lead to conspiracy theory endorsement among those with heightened autistic traits. These results highlight the potential mediating role of systemizing tendencies in the relationship between autistic traits, conspiracy theory endorsement and, based on the results of Study 1, may also apply within this context to scientific reasoning.

## Study 2

Study 1 identified distinct cognitive profiles, particularly in systemizing tendencies, within two latent profiles likely reflecting clinical levels of autistic traits. The findings also suggested that high conspiracy endorsement occurred regardless of scientific reasoning scores within these likely clinical profiles (i.e., Class 3). Therefore, Study 2 aimed to further examine the role of systemizing tendencies in a clinically diagnosed Autism Spectrum Disorder (ASD) population. Specifically, we sought to determine whether systemizing tendencies moderated the relationship between autistic traits and other cognitive factors in predicting conspiracy beliefs.

### Method

#### Participants

As shown in Table 6, the study involved 145 participants from an international panel drawn from *Prolific.Inc* (82 men, 63 women) aged between 18 and 65 years (M = 24.33, SD = 13.20) from a range of countries. All participants had previously received a diagnosis of Autism Spectrum Disorder. Most participants reported that ASD was their sole diagnosis (N = 120) with 22 participants being diagnosed with another mental health condition (12 = Anxiety Disorder, 10 = Anxiety and Depression).

**Table 6:** Demographic characteristics of study 2 (*N* = 145)

	*N* (%)
*Gender*	
Male	82
Female	65
Not stated	0
*Age*	
18–24	53
25–34	54
35–44	22
45–54	10
55–64	6
65+	0
*Country*	
Oceania	12 (8.0)
United Kingdom	33 (22.5)
United States of America	40 (27.5)
Canada	15 (10.5)
South Africa	9 (6.5)
Other continental Europe	26 (18.0)
Rest of the world (nations with, N < 5)	10 (7.0)
*Education*	
University degree	33 (23.0)
Some college	50 (35.0)
High school only	51 (35.0)
Less than high school	12 (8.0)
*Clinical history*	
Received prior diagnosis (co-morbid)	25 (17.0)
ASD only	120 (83.0)
No formal diagnosis	0

#### Sampling procedure

The study was advertised as an investigation into how people view the world and how they interact with social media, with only those diagnosed with Autism Spectrum Disorder included. Participants received monetary compensation for their time and effort (US $7 per hour). All data were anonymised and presented only in group form. Ethical approval was obtained from the Human Research Ethics Subcommittee of Flinders University. An a priori power analysis using *G*Power (v3.1) was conducted for a linear multiple regression testing an interaction effect (R^2^ increase) with one tested predictor (AQ × SQ) and five total predictors (AQ, SQ, AQ × SQ, BADE, SRS). Assuming a medium-to-small effect size (*f*^2^ = 0.08), α = .05, and desired power (1 – β) = 0.80, the required sample size was estimated at *N* = 120. The final sample (*N* = 145) exceeded this threshold, providing sufficient power to detect the hypothesised moderation effect. This expected effect size aligns with conventions in psychological research, where moderation effects are typically small to medium in magnitude (Aguinis et al. 2005). All participants received monetary compensation for their time and effort ($US 7 per hour). The data retrieved was made anonymous and only presented in group form. The study was approved by the Human Research Ethics Subcommittee of Flinders University.

#### Measures

Participants completed the same measures included within *Study 1*: The Systemising Quotient, Autism Quotient, General Conspiracy Belief Scale, Scientific Reasoning Scale and BADE Task*.* Cronbach’s alpha for each ranged between (a = 0.78–0.84) which indicated good internal reliability.

## Results

### Descriptive statistics and correlation analysis

Prior to commencing analysis, one AQ score was found to be an outlier believed to potentially influence on exaggerating effect sizes (i.e., 2.5 below the Standard deviation). Despite some slight deviations from normality on SRS scores, and the obvious slight negative skew of AQ and SQ scores due to being solely a clinical sample, the data distributions were found to be suitable for parametric testing. The majority of participants scored above the midrange for both the AQ and SQ, which is expected due to the sample consisting solely of individuals diagnosed with Autism Spectrum Disorder.

The majority of participants also scored above conventional screening thresholds for both the AQ-10 (cut-off ≥ 6) and SQ-10 (cut-off ≥ 5; Baron-Cohen et al. 2008, 2017), consistent with their verified ASD diagnoses. Fourteen percent of participants (n = 20) scored below the AQ-10 threshold, reflecting known discrepancies between questionnaire cut-offs and formal clinical assessments of autism (Ashwood et al. 2016). Because inclusion was based on a confirmed clinical diagnosis rather than screening criteria, all participants were retained. Sensitivity analysis excluding below-threshold participants produced an equivalent pattern of results (R^2^ = 0.24, ΔR^2^ = 0.01), and no change in the direction or significance of any covariate effects in later analysis. Influence diagnostics indicated that the single AQ outlier did not materially affect the regression results, and the case was therefore kept in all analyses.

A one-way ANOVA revealed no significant differences in conspiracy theory beliefs or systemizing tendencies based on age, gender, or level of education. Table 6 summarizes the descriptive statistics for the different psychometric variables of Study 2. The majority of participants scored above the midrange for both the AQ and SQ, which is expected due to the sample consisting solely of individuals diagnosed with Autism Spectrum Disorder. The sample also scored above the midpoint on the measure of conspiracy theory beliefs (i.e., GCBS scores) (Table 7).

**Table 7:** Descriptive statistics for psychometric measures of study 2

	*M* (*SD*)	Range of participant scores (range)
AQ-10	29.22 (2.88)	26–40 (10–40)
SQ-10	27.30 (4.04)	21–39 (10–40)
*BADE task*		
Liberal acceptance	18.22 (13.70)	0–65 (0–100)
BADE	39.20 (16.52)	− 10–88 (− 100–100)
BACE	43.26 (26.81)	− 10–90 (− 100–100)
SRS	3.26 (3.24)	1–9 (0–11)
GCBS	45.91 (15.33)	15–75 (15–75)

*AQ = 10* Autism quotient-10, *SQ-10* Systemizing quotient-10, *BADE task* Bias against disconfirmatory evidence task, *SRS* Scientific reasoning scale, *GCBS* General conspiracy belief scale

Pearson correlations are presented in Table 8. Consistent with previous research (e.g., Georgiou et al. 2025a, b), there were weak positive correlations between general autistic traits (AQ scores), conspiracy theory endorsement (GCBS scores), and a bias against disconfirmatory evidence (EI scores). Consistent with our hypotheses, systemizing tendencies (SQ scores) showed a positive correlation with general autistic traits (AQ scores) and conspiracy theory endorsement. However, SQ scores also showed a weak positive correlation with both bias against disconfirmatory evidence (BADE) and scientific reasoning performance (SRS scores), while BADE performance showed a negative correlation with scientific reasoning performance. These results suggest a counter-intuitive relationship, wherein higher systemising tendencies were associated with contrasting outcomes: a greater bias against counterevidence, as indicated by BADE performance, but a greater ability to perform scientific reasoning skills, considered to be a robust protective factor against conspiracy beliefs. All correlations remained significant following False Discovery Rate (Benjamini–Hochberg) correction, indicating that the observed relationships were robust to Type I error control. These results also align with the cognitive profiles found in Class 3 of Study 1 (see Table 4).

**Table 8:** Pearson’s correlation between covariates and task performance indices of study 2

	1	2	3	4	5
1. AQ-10					
2. SQ-10	0.406**				
3. SRS	− 0.103	0.108*			
4. GCBS	0.142*	0.103*	− 0.250**		
5. Evidence Integration (BADE)	0.201**	0.084*	− 0.087*	0.230*	

*AQ = 10* Autism quotient-10, *SQ-10* Systemizing quotient-10, *BADE task* Bias against disconfirmatory evidence task, *SRS* Scientific reasoning scale, *GCBS* General conspiracy belief scale, one (*) indicates *p* < 0.05 and two (**) indicate *p* < 0.01

### Moderation regression analysis

Based on the results of Study 1 and preliminary correlations from Study 2, a moderated regression analysis was conducted to examine whether systemizing tendencies (SQ) influence the relationship between general autistic traits (AQ) and conspiracy beliefs (GCBS). BADE and scientific reasoning performance (SRS) were included in the model as covariates. All predictors were mean-centred prior to computing the interaction term to reduce multicollinearity between main effects and interaction terms (Aiken and West 1991).

In Step 1, AQ, SQ, BADE, and SRS were entered as predictors of GCBS. In Step 2, the interaction term (AQ × SQ) was added to test for moderation. The Step 1 model accounted for 19% of the variance in conspiracy theory endorsement (*R*^2^ = 0.19, Adjusted *R*^2^ = 0.17, *F*(4, 140) = 8.21, *p* < 0.001). The addition of the interaction term in Step 2 significantly improved model fit (Δ*R*^2^ = 0.04, Δ*F*(1, 139) = 15.52, *p* < 0.001, *f*^2^ = 0.052), bringing the total explained variance to 23% (*R*^2^ = 0.23, Adjusted *R*^2^ = 0.21). A significant interaction term between systemizing tendencies (SQ) and autistic traits (AQ) on conspiracy belief endorsement was identified (*β* = 0.21, *p* < 0.001), indicating that SQ moderates the relationship between AQ and GCBS. Significant main effects were observed for BADE (β = 0.14, *p* < 0.001) and SRS (*β* = − 0.14, *p* < 0.001). Specifically, higher BADE scores were associated with greater endorsement of conspiracy theories, whereas higher SRS scores were associated with lower endorsement. Full regression coefficients for this model are presented in Table 9.

**Table 9:** Hierarchical moderated regression predicting conspiracy theory endorsement (GCBS)

	*B*	*SE B*	*β*	*t*(*sig.*)
AQ-10	0.11	0.09	0.10	1.22 (0.225)
SQ-10	0.18	0.08	0.17*	2.28 (0.024)
BADE	0.14	0.04	0.14**	3.38 (< 0.001)
SRS	− 0.13	0.04	− 0.14**	−3.26 (< 0.001)
Model 1: R^2^ = 0.19, Adjusted R^2^ = 0.17, *F*(4, 140) = 8.21, *p* < 0.001				
AQ × SQ	0.23	0.06	0.21**	3.94 (< 0.001)
Model 2: *R*^2^ = 0.23, Adjusted *R*^2^ = 0.21, *F*(1, 139) = 15.52, *p* < 0.001, *f*^2^ = 0.052				

*GCBS* General conspiracy belief scale, *AQ* Autism quotient-10, *SQ* Systemizing quotient-10, *SRS* Scientific reasoning scale, *BADE* Bias against disconfirmatory evidence task

To further explore this moderation effect, SQ scores were standardized and categorized into low (−1 SD), average (mean), and high (+1 SD) groups. Conditional-effects analysis revealed that at low levels of SQ, the relationship between AQ and GCBS was weak and non-significant (β = 0.09, *p* = 0.23), whereas at high levels of SQ, the relationship was moderate and significant (β = 0.24, *p* < 0.01). These conditional effects are summarized in Table 10. This finding further emphasises the importance of an individual's cognitive profile, rather than their diagnosis alone (i.e., Roels et al. 2024), for how people may believe in conspiracy theories, as discussed by Georgiou et al. (2024).

**Table 10:** Conditional effects of AQ on GCBS at levels of systemizing tendencies (SQ)

Level of SQ	*B*	*SE*	*Β*	*t*(sig.)
Low (−1 SD)	0.09	0.08	0.09	1.21 (0.23)
Mean (M)	0.16	0.07	0.15	2.30 (0.014)
High (+1 SD)	0.24	0.07	0.24	3.54 (< 0.001)

Dependent variable = GCBS. Conditional effects represent simple slopes of AQ predicting GCBS at different levels of systemizing tendencies (SQ)

## Study 2: Discussion

**Fig. 1:**
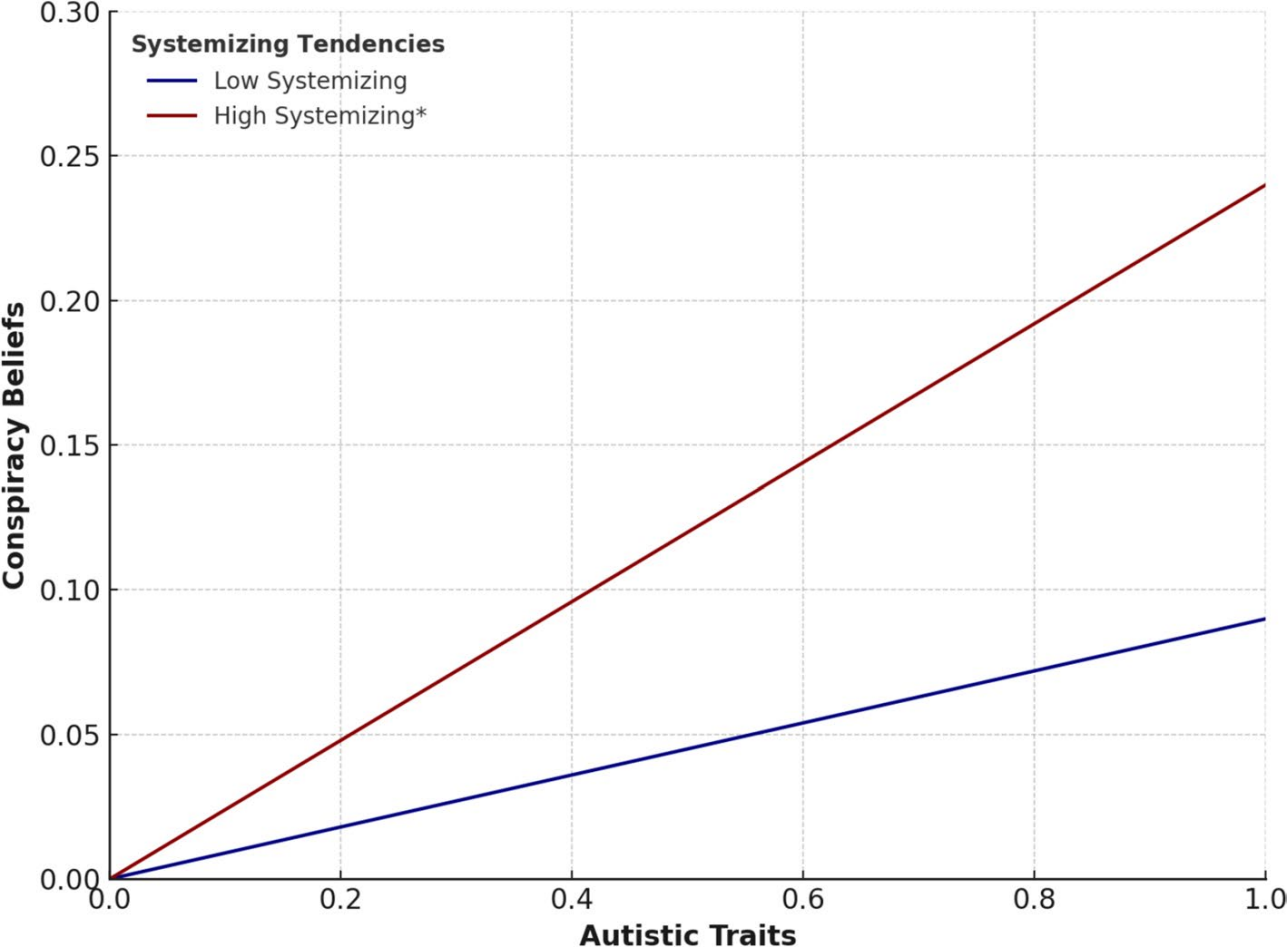
The relationship between autistic traits (AQ) and conspiracy beliefs (GCBS) at low and high levels of systemising tendencies. *Note:* This graph illustrates the moderating effect of SQ on the AQ-GCBS relationship. The asterisk (*) on the High Systemizing line indicates a statistically significant conditional effect. AQ and GCBS are plotted on standardized scales. Systemizing Tendencies (SQ) represent standardized levels at −1 SD (Low) and +1 SD (High).

**Fig. 2:**
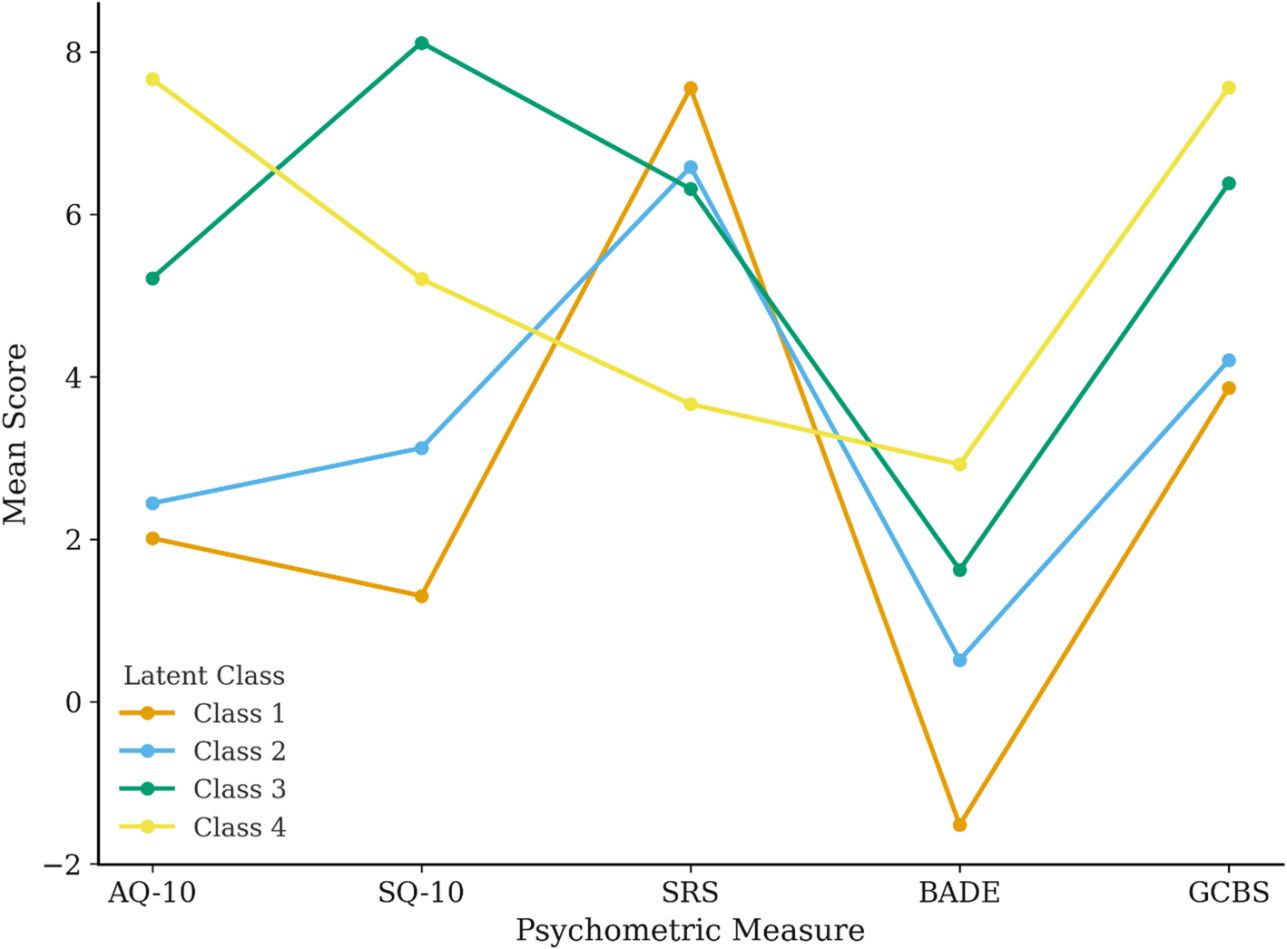
Mean psychometric profiles across four latent classes in study 1. Note: Pattern of mean scores as a function of latent class models across the Autism quotient-10, AQ-10; Systemising quotient-10, SQ; Scientific reasoning scale, SRS; Evidence integration score on the bias against disconfirmatory evidence task, BADE; General conspiracy belief scale, GCBS

The results of Study 2 demonstrate that, within a clinical sample, systemizing tendencies moderate the relationship between autistic traits and conspiracy theory beliefs. In other words, the evidence suggests that autistic traits do not directly promote conspiracy beliefs but may be associated with a hyper-systemized approach to processing information. This hyper-systemized approach appears to underlie individual differences related to potentially conspiracy beliefs and cognitive biases. As shown in Fig. 1, Systemizing tendencies significantly amplified the relationship between autistic traits and conspiracy beliefs at high levels of SQ but not at low levels, underscoring the moderating role of systemizing tendencies. Moreover, belief inflexibility, as measured by BADE scores, emerged as an important contributor. BADE scores, combined with SQ, jointly predicted conspiracy beliefs, supporting the notion that cognitive rigidity driven by systemizing tendencies plays a critical role in conspiracy theory endorsement (Fig. 2).

## General discussion

The aim of this study was to investigate the predictive value of systemizing tendencies in explaining the relationship between autistic traits and conspiracy theory beliefs. More broadly, it sought to evaluate whether these findings support the hyper-systemizing hypothesis, which proposes that a preference for systematic and internally consistent thinking may contribute to the development of conspiracy beliefs. Study 1, conducted in a general population sample, identified a distinct cognitive profile (Class 3), in which individuals with high systemizing tendencies exhibited greater endorsement of conspiracy beliefs and belief inflexibility, despite demonstrating scientific reasoning ability comparable to other groups. While scientific reasoning is generally considered a robust indicator of reasoning ability and a known protective factor against conspiracy beliefs, its role in this study appeared more contextual. Specifically, higher systemizing tendencies and autistic traits were associated with conspiracy beliefs regardless of scientific reasoning ability. Study 2 further demonstrated that systemizing tendencies moderated the relationship between autistic traits and conspiracy beliefs within a clinical sample.

Overall, these findings contribute to the broader discussion on how reasoning abilities relate to conspiracy beliefs, suggesting that systemizing tendencies may influence belief persistence, irrespective of scientific reasoning skills. They also provide a more nuanced understanding of the role of autistic traits in conspiracy endorsement. The findings centred around systemising tendencies also highlight the importance of context in cognitive explanatory models of conspiracy beliefs.

### Implications for conspiracy research: an inclusive framework

The finding that systemizing tendencies are associated with both scientific reasoning (a known barrier to conspiracy beliefs) and conspiracy belief endorsement underscores the need for a more integrated research framework that extends beyond existing cognitive models. While some integrated models have been proposed to examine the antecedents of conspiracy beliefs—either through comparisons of predictors (e.g., Georgiou et al. 2019) or cross-examinations via reviews (e.g., Bowes et al. 2021; Biddestone et al. 2022; Goreis and Voracek 2019)—the statistical methods used to analyse conspiracy beliefs still largely assume that reasoning ability is the primary determinant (Pilch et al. 2023).

For instance, whether through Bayesian modelling, signal detection analyses, or frequentist methods such as those in this study (e.g., latent profiles), most approaches implicitly frame belief formation as a matter of distinguishing truth from falsehood or assessing how reasoning and thinking styles affect belief accuracy. However, they often overlook whether conspiracy believers prioritize accuracy in the first place. Douglas and Sutton (2023, 2024) have also raised concerns about conspiracy belief outcome measures, noting that ‘endorsement’ is typically framed in terms of accuracy rather than accounting for broader motivational influences. This study highlights this issue by demonstrating that systemizing tendencies predict conspiracy beliefs independently of reasoning ability, reinforcing the need to consider alternative frameworks. While current measurement approaches fail to account for how motivational and identity-based factors interact with cognitive tendencies, motivated reasoning provides a potential lens for future research. This conceptual area remains largely unexplored, but the hyper-systemizing hypothesis could help examine how these factors collectively shape belief formation. However, a clearer framework is needed to effectively integrate these perspectives.

### Implications for autism research and intervention

The findings of Georgiou et al. (2024), alongside the present study on systemizing tendencies, suggest that engagement with conspiracy content may persist regardless of reasoning ability when an individual has a strong preference for systemizing within an autistic population. This has implications for research that has primarily examined conspiracy beliefs in autistic populations through diagnostic group comparisons without incorporating broader cognitive profiles. Roels et al. (2024) examined group-level differences in conspiracy beliefs but did not account for systemizing tendencies or other cognitive factors that may interact with belief formation. The present findings indicate that within-group variability, particularly in cognitive style, may be key to understanding why some autistic individuals are more prone to conspiracy beliefs. Rather than focusing solely on autism as a diagnostic category, future research should consider the associated traits—such as systemizing tendencies and specific cognitive profiles—that may drive engagement with conspiracy content.

More broadly, these results highlight the risks autistic individuals may face in digital media spaces and raise questions about how to address conspiracy theory engagement in potentially vulnerable groups. If engagement is driven by a preference for systemizing rather than an ability to discern truth based on reasoning, the common strategy of providing an "inoculation" against conspiracy content may be ineffective. Many existing interventions focus on countering the validity of conspiracy claims but fail to address their motivational purpose or alternative functions. Indeed, Williams et al. (2024a) has emphasized that people rarely change their conspiracy beliefs over time, suggesting that current intervention methods insufficiently account for factors beyond logic and reasoning. This may explain the weak effect sizes often found in studies testing interventions for conspiracy beliefs in autistic samples (e.g., Georgiou et al. 2023, 2025).

Therefore, future research should explore alternative intervention approaches tailored to individuals with high systemizing tendencies. In particular, qualitative research is needed to better understand the motivations underlying engagement with conspiracy content in autistic individuals. This may provide insights into how interventions can address not only accuracy but also the broader appeal and function of such beliefs.

## Limitations

While the sample included both general and autistic populations, autism diagnoses were self-reported via the international sampling panel *Prolific.co*. Therefore, scoring above the cut-off for clinically significant traits does not equate to a formal clinical diagnosis. Another limitation is the use of international online panels, as participants tend to be more highly educated than the general population (Blasius et al. 2010). A further limitation of the individual differences approach used in this study is that it does not account for how the examined factors influence misbeliefs that arise at the group level (e.g., social influence; Thomas et al. 2024). Future research could explore how individuals with higher autistic traits, stronger systemizing tendencies engage with conspiracy theories in group-based contexts (e.g., simulated chat forums or linguistic analysis; Biddlestone et al. 2022; Thomas et al. 2024; Robertson et al. 2022; van Prooijen 2024). Moreover, the level of endorsement for misinformation or conspiracy theories in this study may not fully reflect participants' underlying misbeliefs about the world without repeated or longitudinal assessments to track how these beliefs manifest across different contexts. As with similar research in this area, it is also important to acknowledge that the findings were drawn from a convenience sample.

## Conclusions

The findings of this study support the hyper-systemizing hypothesis, demonstrating that systemizing tendencies, rather than autistic traits alone, contribute to conspiracy endorsement. Across both general and autistic populations, individuals with high systemizing tendencies were more likely to endorse conspiracy theories, suggesting that belief formation is not solely driven by deficits in reasoning but by a preference for structured, internally consistent explanations. This research contributes more broadly to understanding how cognitive styles, particularly systemizing, shape belief formation beyond traditional reasoning deficits. By highlighting the role of systemizing tendencies in conspiracy belief endorsement, these findings suggest that interventions should move beyond logic- and reasoning-based approaches to account for cognitive preferences and motivational factors.

## Supplementary Information

Below is the link to the electronic supplementary material.

Supplementary file1 (DOCX 31 kb)

## References

[CR2] Aguinis H, Beaty JC, Boik RJ, Pierce CA (2005) Effect size and power in assessing moderating effects of categorical variables using multiple regression: a 30-year review. J Appl Psychol 90(1):94–107 15641892 10.1037/0021-9010.90.1.94

[CR77] Aiken LS, West SG (1991) Multiple regression: Testing and interpreting interactions. Sage Publications, Inc.

[CR75] Akaike H (1987) Factor analysis and AIC. Psychometrika 52(3):317–332

[CR1] Ashwood KL, Gillan N, Horder J, Hayward H, Woodhouse E, McEwen FS, Findon J, Eklund H, Spain D, Wilson CE, Cadman T, Young S, Stoencheva V, Murphy C, Robertson D, Charman T, Bolton P, Glaser K, Asherson P, Simonoff E, Murphy DG (2016) Predicting the diagnosis of autism in adults using the Autism-Spectrum Quotient (AQ) questionnaire. Psychol Med 46(12):2595–2604 27353452 10.1017/S0033291716001082 PMC4988267

[CR12] Čavojová V, Šrol J, Ballová Mikušková E (2020) How scientific reasoning correlates with health-related beliefs and behaviors during the COVID-19 pandemic? J Health Psychol 27(3):534–547 33016131 10.1177/1359105320962266

[CR13] Čavojová V, Šrol J, Jurkovič M (2020) Why should we try to think like scientists? The role of scientific reasoning in susceptibility to epistemically suspect beliefs and cognitive biases. Appl Cogn Psychol 34(1):85–95

[CR65] Ballová Mikušková E, Čavojová V (2020) The effect of analytic cognitive style on credulity. Front Psychol 11:584424 10.3389/fpsyg.2020.584424 PMC7593255 33178085

[CR4] Baron-Cohen S (2008) Autism, hyper systemizing, and truth. Q J Exp Psychol 61(1):64–75 10.1080/17470210701508749 18038339

[CR3] Baron-Cohen S (2017) Editorial perspective: neurodiversity - a revolutionary concept for autism and psychiatry. J Child Psychol Psychiatry Allied Discip 58(6):744–747 10.1111/jcpp.12703 28524462

[CR5] Berlin KS, Williams NA, Parra GR (2014) An introduction to latent variable mixture modelling (Part 1): overview and cross-sectional latent class and latent profile analyses. J Pediatr Psychol 39(2):174–187. 10.1093/jpepsy/jst084 24277769 10.1093/jpepsy/jst084

[CR6] Biddlestone M, Green R, Cichocka A, Douglas K, Sutton R (2022) A systematic review and meta-analytic synthesis of the motives associated with conspiracy beliefs 10.1037/bul0000463 39913483

[CR7] Blasius J, Brandt M (2010) Representativeness in online surveys through stratified samples. Bull Sociol Methodol 107(1):5–21

[CR8] Bowes SM, Costello TH, Ma W, Lilienfeld SO (2021) Looking under the tinfoil hat: clarifying the personological and psychopathological correlates of conspiracy beliefs. J Pers 89(3):422–436 32852781 10.1111/jopy.12588

[CR9] Brosnan M, Ashwin C (2023) Thinking, fast and slow on the autism spectrum. Autism 27(5):1245–1255 36325717 10.1177/13623613221132437 PMC10291371

[CR10] Brotherton R, French CC, Pickering AD (2013) Measuring belief in conspiracy theories: the generic conspiracist beliefs scale. Front Psychol 4:279 23734136 10.3389/fpsyg.2013.00279 PMC3659314

[CR11] Caldwell-Harris CL, Jordan CJ (2014) Systemizing and special interests: characterizing the continuum from neurotypical to autism spectrum disorder. Learn Individ Differ 29:98–105

[CR14] De Coninck D, Frissen T, Matthijs K, d’Haenens L, Lits G, Champagne-Poirier O, Généreux M (2021) Beliefs in conspiracy theories and misinformation about COVID-19: comparative perspectives on the role of anxiety, depression and exposure to and trust in information sources. Front Psychol 12:646394 33935904 10.3389/fpsyg.2021.646394 PMC8085263

[CR15] Deleuze J, Rochat L, Romo L, Van der Linden M, Achab S, Thorens G et al (2015) Prevalence and characteristics of addictive behaviors in a community sample: a latent class analysis. Addict Behav Rep 1:49–56 29531979 10.1016/j.abrep.2015.04.001 PMC5845955

[CR16] Denovan A, Dagnall N, Drinkwater K, Parker A (2018) Latent profile analysis of schizotypy and paranormal belief: associations with probabilistic reasoning performance. Front Psychol 9:35 29434562 10.3389/fpsyg.2018.00035 PMC5791384

[CR20] Douglas KM, Sutton RM (2023) What are conspiracy theories? A definitional approach to their correlates, consequences, and communication. Annu Rev Psychol 74(1):271–298 36170672 10.1146/annurev-psych-032420-031329

[CR19] Douglas KM, Sutton RM, Cichocka A (2017) The psychology of conspiracy theories. Curr Dir Psychol Sci 26(6):538–542. 10.1177/0963721417718261 29276345 10.1177/0963721417718261 PMC5724570

[CR18] Douglas KM, Uscinski JE, Sutton RM, Cichocka A, Nefes T, Ang CS, Deravi F (2019) Understanding conspiracy theories. Polit Psychol 40(S1):3–35

[CR17] Douglas K, Sutton RM (2024) The social psychology of conspiracy theories: key insights and future challenges. Adv Exp Soc Psychol

[CR21] Drummond C, Fischhoff B (2017) Development and validation of the scientific reasoning scale. J Behav Decis Mak 30:26–38

[CR23] Georgiou M, Delfabbro PH, Balzan R (2019) Conspiracy beliefs in the general population: the importance of psychopathology, cognitive style and educational attainment. Pers Individ Differ 151:109521

[CR24] Georgiou M, Delfabbro PH, Balzan R (2020) COVID-19-related conspiracy beliefs and their relationship with perceived stress and pre-existing conspiracy beliefs. Pers Individ Differ 166:110201 10.1016/j.paid.2020.110201 PMC7296298 32565592

[CR69] Georgiou N, Balzan RP, Delfabbro P, Young R (2024) People with autistic traits are more likely to engage with misinformation and conspiracy theories in a simulated social media context. Cogn Neuropsychiatry 29(4–5):286–305 10.1080/13546805.2024.2443576 39718936

[CR22] Georgiou N, Balzan RP, Delfabbro P, Young R (2024) People with autistic traits are more likely to engage with misinformation and conspiracy theories in a simulated social media context. Cogn Neuropsychiatry 29(4–5):286–305. 10.1080/13546805.2024.2443576 39718936 10.1080/13546805.2024.2443576

[CR25] Georgiou N, Delfabbro P, Balzan R (2021) Autistic traits as a potential confounding factor in the relationship between schizotypy and conspiracy beliefs. Cogn Neuropsychiatry 26(4):273–292 33970807 10.1080/13546805.2021.1924650

[CR27] Georgiou N, Delfabbro P, Balzan R (2021) Conspiracy-beliefs and receptivity to disconfirmatory information: a study using the BADE task. SAGE Open 1(11):1–9

[CR28] Georgiou N, Delfabbro P, Balzan R (2021) Conspiracy theory beliefs, scientific reasoning and the analytical thinking paradox. Appl Cogn Psychol 35(6):1523–1543

[CR26] Georgiou N, Delfabbro P, Balzan R (2021) Could autistic traits be a risk factor for conspiracy beliefs? An analysis of cognitive style and information-seeking behaviour. Minerva Psychiatry 62(4):231–240

[CR67] Georgiou N, Delfabbro P, Balzan R (2022) Latent profile analysis of schizotypy, autistic traits and conspiracy theory beliefs: Associations with cognitive flexibility and scientific reasoning performance. J Experimental Psychopathol 13(3):20438087221125046

[CR68] Georgiou N, Delfabbro P, Balzan R (2023) The effectiveness of a scientific reasoning intervention for conspiracy theory beliefs. Appl Cogn Psychol 37(2):369–382

[CR29] Georgiou N, Moritz S, Woodward T, Delfabbro P, Balzan R (2025) Correcting the bias in pseudoscience: a pilot of the cognitive bias correction intervention to improve evidence interpretation. J Cogn Psychol

[CR30] Georgiou N, Woodward T, Moritz S, Menon M, Balzan R (2025) Measuring belief flexibility in autism: an evaluation of the BADE task scoring methods with autistic respondents. J Exp Psychopathol

[CR31] Gjoneska B (2021) Conspiratorial beliefs and cognitive styles: an integrated look on analytic thinking, critical thinking, and scientific reasoning in relation to (dis)trust in conspiracy theories. Front Psychol 12:736838 34712182 10.3389/fpsyg.2021.736838 PMC8545864

[CR32] Goreis A, Voracek M (2019) A systematic review and meta-analysis of psychological research on conspiracy beliefs: field characteristics, measurement instruments, and associations with personality traits. Front Psychol 10:205 30853921 10.3389/fpsyg.2019.00205 PMC6396711

[CR73] Greenberg DM, Warrier V, Allison C, Baron-Cohen S (2018) Testing the Empathizing-Systemizing theory of sex differences and the Extreme Male Brain theory of autism in half a million people. Proc Natl Acad Sci USA 115(48):12152–12157 10.1073/pnas.1811032115 PMC6275492 30420503

[CR71] Hoekstra RA, Vinkhuyzen AA, Wheelwright S, Bartels M, Boomsma DI, Baron-Cohen S, Van, Der Sluis S (2011) The construction and validation of an abridged version of the autism-spectrum quotient (AQ-Short). J Autism Dev Disord 41(5):589–596 10.1007/s10803-010-1073-0 PMC3076581 20697795

[CR34] Hussain Z, Williams GA, Griffiths MD (2015) An exploratory study of the association between online gaming addiction and enjoyment motivations for playing massively multiplayer online role-playing games. Comput Human Behav 50:221–230

[CR35] Jolley D, Marques MD, Cookson D (2022) Shining a spotlight on the dangerous consequences of conspiracy theories. Curr Opin Psychol 47:101363 35732091 10.1016/j.copsyc.2022.101363 PMC9142208

[CR36] Lantian A, Bagneux V, Delouvée S, Gauvrit N (2021) Maybe a free thinker but not a critical one: high conspiracy belief is associated with low critical thinking ability. Appl Cogn Psychol 35(3):674–684

[CR76] Lo Y, Mendell NR, Rubin DB (2001) Testing the number of components in a normal mixture. Biometrika 88(3):767–778

[CR37] Masyn KE (2013) Latent class analysis and finite mixture modeling. In: Little TD (ed), The Oxford handbook of quantitative methods: statistical analysis. vol 2, pp 551–611. Oxford University Press

[CR38] Nera K, Douglas KM, Bertin P, Delouvée S, Klein O (2024) Conspiracy beliefs and the perception of intergroup inequalities. Pers Soc Psychol Bull 01461672241279085 10.1177/01461672241279085 39323225

[CR40] Norris P, Epstein S (2011) An experiential thinking style: its facets and relations with objective and subjective criterion measures. J Pers 79(5):1043–1079 21241307 10.1111/j.1467-6494.2011.00718.x

[CR39] Nylund KL, Asparouhov T, Muthén BO (2007) Deciding on the number of classes in latent class analysis and growth mixture modeling: a Monte Carlo simulation study. Struct Equ Model 14(4):535–569. 10.1080/10705510701575396

[CR41] Park HO (2023) Autism Spectrum Disorder and Savant Syndrome: a systematic literature review. J Korean Acad Child Adolesc Psychiatry 34(2):76–92 37035789 10.5765/jkacap.230003 PMC10080257

[CR45] Pennycook G, Epstein Z, Mosleh M, Arechar AA, Eckles D, Rand DG (2021) Shifting attention to accuracy can reduce misinformation online. Nature 592(7855):590–595 33731933 10.1038/s41586-021-03344-2

[CR42] Pennycook G, Epstein Z, Mosleh M, Arechar A, Eckles D, Rand D (2021) Shifting attention to accuracy can reduce misinformation online. Nature 592:590–595 33731933 10.1038/s41586-021-03344-2

[CR43] Pennycook G, Rand DG (2020) Who falls for fake news? The roles of bullshit receptivity, overclaiming, familiarity, and analytic thinking. J Pers 88(2):185–200 30929263 10.1111/jopy.12476

[CR44] Pennycook G, Rand DG (2021) The psychology of fake news. Trends Cogn Sci 25(5):388–402 33736957 10.1016/j.tics.2021.02.007

[CR46] Pennycook G, Rand DG (2022) Accuracy prompts are a replicable and generalizable approach for reducing the spread of misinformation. Nat Commun 13(1):2333 35484277 10.1038/s41467-022-30073-5 PMC9051116

[CR47] Pilch I, Turska-Kawa A, Wardawy P, Olszanecka-Marmola A, Smołkowska-Jędo W (2023) Contemporary trends in psychological research on conspiracy beliefs. A systematic review. Front Psychol 14:1075779 36844318 10.3389/fpsyg.2023.1075779 PMC9945548

[CR48] Pytlik N, Soll D, Mehl S (2020) Thinking preferences and conspiracy belief: intuitive thinking and the jumping to conclusions-bias as a basis for the belief in conspiracy theories. Front Psychiatry 11:568942 33061922 10.3389/fpsyt.2020.568942 PMC7530244

[CR49] Ramaswamy V, DeSarbo WS, Reibstein DJ, Robinson WT (1993) An empirical pooling approach for estimating marketing mix elasticities with PIMS data. Marketscience 12:103–124

[CR50] Robertson CE, Pretus C, Rathje S, Harris E, Van Bavel JJ (2022) How social identity shapes conspiratorial belief. Curr Opin Psychol. 10.1016/j.copsyc.2022.101423 35987090 10.1016/j.copsyc.2022.101423

[CR66] Roberts R, Risen JL (2022) Introducing conspiracy intuitions to better understand conspiracy beliefs. Curr Opin Psychol 47:101395 10.1016/j.copsyc.2022.101395 35842985

[CR51] Roels S, Begeer S, Scheeren AM, van Prooijen JW (2024) Conspiracy mentality in autistic and non-autistic individuals. Cogn Neuropsychiatry, 1–14 10.1080/13546805.2024.2399505 PMC11750142 39254641

[CR53] Sanford N, Veckenstedt R, Moritz S, Balzan RP, Woodward TS (2014) Impaired integration of disambiguating evidence in delusional schizophrenia patients. Psychol Med 44(13):2729–2738 25065271 10.1017/S0033291714000397

[CR52] Schwarz G (1978) Estimating the dimension of a model. Open J Stat 6:461–464

[CR54] Sclove SL (1987) Application of model-selection criteria to some problems in multivariate analysis. Psychometrika 52:333–343

[CR55] Sindermann C, Cooper A, Montag C (2019) Empathy, autistic tendencies, and systemizing tendencies—relationships between standard self-report measures. Front Psychiatry 10:307 31143133 10.3389/fpsyt.2019.00307 PMC6522547

[CR70] Stevenson JL, Hart KR (2017) Psychometric properties of the autism-spectrum quotient for assessing low and high levels of autistic traits in college students. J Autism Dev Disord 47(6):1838–1853 10.1007/s10803-017-3109-1 28349365

[CR56] Ståhl T, Van Prooijen JW (2018) Epistemic rationality: skepticism toward unfounded beliefs requires sufficient cognitive ability and motivation to be rational. Pers Individ Differ 122:155–163

[CR57] Swami V, Barron D, Weis L, Voracek M, Stieger S, Furnham A (2017) An examination of the factorial and convergent validity of four measures of conspiracist ideation, with recommendations for researchers. PLOS ONE 12:e0172617 28231266 10.1371/journal.pone.0172617 PMC5322923

[CR58] Swami V, Furnham A, Smyth N, Weis L, Lay A, Clow A (2016) Putting the stress on conspiracy theories: examining associations between psychological stress, anxiety, and belief in conspiracy theories. Pers Ind Diff 99:72–76

[CR59] Thomas EF, Bird L, O’Donnell A, Osborne D, Buonaiuto E, Yip L, Lizzio-Wilson M, Wenzel M, Skitka L (2024) Do conspiracy beliefs fuel support for reactionary social movements? Effects of misbeliefs on actions to oppose lockdown and to “stop the steal.” Br J Soc Psychol. 10.1111/bjso.12727 38314917 10.1111/bjso.12727

[CR60] Uscinski J, Enders A, Klofstad C, Seelig M, Drochon H, Premaratne K, Murthi M (2022) Have beliefs in conspiracy theories increased over time? PLoS One 17(7):e0270429 35857743 10.1371/journal.pone.0270429 PMC9299316

[CR61] van Prooijen JW (2024) Group-oriented motivations underlying conspiracy theories. Group Processes Intergroup Relations, 13684302241240696

[CR72] Wheelwright S, Baron-Cohen S, Goldenfeld N, Delaney J, Fine D, Smith R, Weil L, Wakabayashi A (2006) Predicting Autism Spectrum Quotient (AQ) from the Systemizing Quotient-Revised (SQ-R) and Empathy Quotient (EQ). Brain Res 1079(1):47–56 10.1016/j.brainres.2006.01.012 16473340

[CR62] Williams DL, Mazefsky CA, Walker JD, Minshew NJ, Goldstein G (2014) Associations between conceptual reasoning, problem solving, and adaptive ability in high-functioning autism. J Autism Dev Disord 44(11):2908–2920 25099486 10.1007/s10803-014-2190-y PMC6067678

[CR63] Williams MN, Marques MD, Kerr JR, Hill SR, Ling M, Clarke EJR (2024) Increased belief in one conspiracy theory leads to increased belief in others over time. 10.31234/osf.io/qpgvh

[CR74] Woodward TS, Moritz S, Cuttler C, Whitman JC (2006) The contribution of a cognitive bias against disconfirmatory evidence (BADE) to delusions in schizophrenia. J Clin Exp Neuropsychol 28(4):605–617 10.1080/13803390590949511 16624787

[CR64] Zeng J, Schäfer MS, Oliveira TM (2022) Conspiracy theories in digital environments: moving the research field forward. Converg Int J Res New Media Technol 28(4):929–939 10.1177/13548565221117474 PMC9483695 36147519

