# Supplementary material for: The hyper-systemizing hypothesis: how the tendency to systemize influences conspiracy beliefs and belief inflexibility in clinical and general populations
Source: Cogn Process. 2026 Jan 14;27(2):433–45. doi: 10.1007/s10339-025-01326-0 (PMC13156087; doi:10.1007/s10339-025-01326-0)
Supplement: Supplementary file 1 — Supplementary file1 (DOCX 31 kb) [file 10339_2025_1326_MOESM1_ESM.docx]

**Appendices for Review**

| **Measure** | **Number of Items** | **Typical response format** | **Reference** | **Item list included? (Y/N)** |
| --- | --- | --- | --- | --- |
| AQ-10 | 10 | 4-point Likert (Agree–Disagree) | Baron-Cohen et al. (2001) | Y |
| SQ-10 | 10 | 4-point Likert (Strongly Agree–Strongly Disagree) | Greenberg, Warrier & Baron-Cohen (2018) | Y |
| SRS | 11 | Multiple choice / True‐False | Drummond & Fischhoff (2017) | Partially |
| GCBS | 15 | 5-point Likert (1 = Not True, 5 = True) | Brotherton et al. (2013) | Partially |
| BADE | Task paradigm (various items/stimuli) | Ratings of plausibility across evidence steps | Woodward (2007) et al. | N |
|  |  |  |  |  |

**Autism Spectrum Quotient – 10 (AQ-10)**

**AQ-10_for_adults**

**Instructions:** Please tick one option per question only.
(Responses: *Definitely agree / Slightly agree / Slightly disagree / Definitely disagree*)

1. I often notice small sounds when others do not.
2. I usually concentrate more on the whole picture, rather than the small details.
3. I find it easy to do more than one thing at once.
4. If there is an interruption, I can switch back to what I was doing very quickly.
5. I find it easy to “read between the lines” when someone is talking to me.
6. I know how to tell if someone listening to me is getting bored.
7. When I’m reading a story, I find it difficult to work out the characters’ intentions.
8. I like to collect information about categories of things (e.g., types of car, bird, train, or plant).
9. I find it easy to work out what someone is thinking or feeling just by looking at their face.
10. I find it difficult to work out people’s intentions.

**Scoring:**
Score one point for “definitely” or “slightly agree” on items 1, 7, 8, and 10.
Score one point for “definitely” or “slightly disagree” on items 2, 3, 4, 5, 6, and 9.
A total score ≥ 6 indicates that an autism assessment may be warranted.

**Reference:**
Baron-Cohen, S., Wheelwright, S., Skinner, R., Martin, J., & Clubley, E. (2001). *The Autism Spectrum Quotient (AQ): Evidence from Asperger Syndrome/High-Functioning Autism, Males and Females, Scientists and Mathematicians.* *Journal of Autism and Developmental Disorders, 31*(1), 5–17.
(Adult AQ-10: Autism Research Centre, University of Cambridge.)

**Systemizing Quotient – Revised (SQ-R-10)**

**SQ10-Adult**

**Instructions:** Read each statement carefully and rate how strongly you agree or disagree.
(Responses: *Strongly agree / Slightly agree / Slightly disagree / Strongly disagree*)

1. When I learn about a new category, I like to go into detail to understand the small differences between its members.
2. When I’m in a plane, I do not think about the aerodynamics.
3. I am interested in knowing the path a river takes from its source to the sea.
4. When travelling by train, I often wonder exactly how the rail networks are coordinated.
5. When I hear the weather forecast, I am not very interested in the meteorological patterns.
6. I enjoy looking through catalogues of products to see the details of each product and how it compares to others.
7. When I look at a mountain, I think about how precisely it was formed.
8. When I look at a piece of furniture, I do not notice the details of how it was constructed.
9. When I learn a language, I become intrigued by its grammatical rules.
10. When I listen to a piece of music, I always notice the way it’s structured.

**Scoring:**

- Score 2 points for “definitely agree,” 1 point for “slightly agree,” and 0 for disagreeing responses on items 1, 3, 4, 6, 7, 9, 10.
- Score 2 points for “strongly disagree,” 1 for “slightly disagree,” and 0 for agreeing responses on items 2, 5, 8.

**Reference:**
Greenberg, D. M., Warrier, V., Allison, C., & Baron-Cohen, S. (2018). *Testing the Empathizing-Systemizing Theory of Sex Differences and the Extreme Male Brain Theory of Autism in Half a Million People.* *Proceedings of the National Academy of Sciences, 115*(48), 12152–12157. https://doi.org/10.1073/pnas.1811032115

**Scientific Reasoning Scale (SRS)**

*(Drummond & Fischhoff, 2017)*

**Instructions:**
Read each scenario and indicate whether the researcher’s conclusion or action is correct (*True*) or incorrect (*False*). Options are True, False and Don’t Know.

| **#** | **Concept Tested** | **Item** | **Correct Answer** | | |
| --- | --- | --- | --- | --- | --- |
| 1 | Blind/double blind | In a taste test, a researcher puts Brand A coffee in a cup with white tape on it and Brand B coffee in an identical cup with black tape on it. A lab assistant gives tasters one of the cups, while the researcher watches their facial expressions. True or False? The lab assistant should not watch the cups being filled. | | **True** |  |
| 2 | Causality | A researcher finds that American states with larger parks have fewer endangered species. True or False? These data show that increasing the size of American state parks will reduce the number of endangered species. | | **False** |  |
| 3 | Confounding variables | A researcher has subjects put together a jigsaw puzzle either in a cold room with a loud radio or in a warm room with no radio. Subjects solve the puzzle more quickly in the warm room with no radio. True or False? The scientist cannot tell if the radio caused subjects to solve the puzzle more slowly. | | **True** |  |
| 4 | Construct validity | An education researcher wants to measure the general math ability of a sample of high-performing math students. All the students have taken classes in geometry and pre-calculus. True or False? The education researcher can measure general math ability by giving the students a geometry test. | | **False** |  |
| 5 | Control group | Two scientists test an anti-acne cream on teenagers with acne. Scientist A wants to give the cream to all the teenagers in the study. Scientist B wants to give the cream to half the teenagers and give the cream without anti-acne ingredients to the other half. True or False? Both ways of testing the cream are equally good. | | **False** |  |
| 6 | Ecological validity | A researcher has a group of subjects play a competitive game. Each subject’s goal is to make money by buying and selling tokens. Subjects are paid a flat fee for participating in the experiment. True or False? The researcher can confidently state that the behavior in the experiment reflects real-life buying and selling behavior. | | **False** |  |
| 7 | History | A randomly selected sample of Americans is surveyed about Disease A before and after a 6-month media campaign about the disease. Midway through the campaign, a celebrity dies of Disease A. The survey data indicate that knowledge of Disease A is higher after the campaign. True or False? The media campaign may not have increased knowledge of Disease A. | | **True** |  |
| 8 | Maturation | Subjects in an experiment must press a button whenever a blue dot flashes on their computer screen. At first, the task is easy for subjects. But as they continue, they make more and more errors. True or False? The blue dot must flash more quickly as the task progresses. | | **False** |  |
| 9 | Random assignment to condition | Researchers want to see whether a health intervention helps school children lose weight. School children are sorted into either an intervention or control group. True or False? The researchers should assign the overweight children to the intervention group. | | **False** |  |
| 10 | Reliability | A researcher develops a new method for measuring the surface tension of liquids. This method is more consistent than the old method. True or False? The new method must also be more accurate than the old method. | | **False** |  |
| 11 | Response Bias | Two researchers are developing a survey to measure consumers’ feelings about customer service. Researcher A wants customers to rate their agreement with the statement “I am satisfied with customer service” on a 5-point scale (1 = strongly agree to 5 = strongly disagree). Researcher B wants customers to rate customer service on a 5-point scale (1 = not dissatisfied at all to 5 = highly dissatisfied). True or False? These questions are the same. | | **False** | |
|  |  |  |  |  |  |

**The Bias Against Disconfirmatory Evidence Task (Woodward et al., 2006)**

The Items listed below are BADE items taken from the inventory of Woodward et al. 2007 – not for distribution.

**TRIAL 1**

STATEMENTS

"Judy saved the little girl's life."

"Judy's family supported Judy's decision to help the little girl."

"Luckily, Judy is the same blood-type as the little girl."

INTERPRETATIONS

"Judy is a fire-fighter." NL

"Judy is a doctor." EL

"Judy is a witch." A

"Judy is an organ donor." T

**TRIAL 2**

STATEMENTS

"The picnic came to an abrupt end."

"Everyone went home stunned from the picnic's outcome."

"By the time the ambulance arrived it was too late."

INTERPRETATIONS

"It started to rain unexpectedly." NL

"The families began to argue with one another." EL

"The volcano began to erupt." A

"Audrey died from an allergic reaction to a bee sting." T

**TRIAL 3**

STATEMENTS

"Sometimes Veronica goes home in tears."

"Veronica does not have many friends."

"Veronica is afraid of her classmates."

INTERPRETATIONS

"Veronica is always overly dramatic." NL

"Veronica is suffering from the side effects of her medication." EL

"Veronica likes to water the plants with her tears." A

"Veronica is bullied at school." T

**TRIAL 4**

STATEMENTS

"Susan can hardly speak or think."

"Susan made a bad decision in her past."

"Susan must begin taking her medication immediately."

INTERPRETATIONS

"Susan is very drunk." NL

"Susan has a mental disability." EL

"Susan does not like doing much." A

"Susan has just been diagnosed with HIV." T

**TRIAL 5**

STATEMENTS

"Sandra works downtown."

"Sandra always works alone."

"Sandra's clients are only men."

INTERPRETATIONS

"Sandra is a lawyer in a large firm." NL

"Sandra works in a soup-kitchen for the homeless." EL

"Sandra is afraid of the countryside." A

"Sandra is a prostitute." T

**TRIAL 6**

STATEMENTS

"Mark often comes home late from work."

"Mark tells his wife that he is not in the mood to spend time with her."

"Mark's wife is suspicious."

INTERPRETATIONS

"Mark is trying to earn a promotion at work." NL

"Mark must work hard in order to not lose his job." EL

"Mark thinks that money grows on trees." A

"Mark is having an affair." T

**Generic Conspiracist Belief Scale Revision (Brotherton et al., 2013)**

This brief survey is designed to assess your beliefs about some of these subjects. Please indicate the degree to which you believe each statement is likely to be true on the following scale: Definitely not true; Probably not true; Not sure/cannot decide; Probably true; Definitely true

1. The government is involved in the murder of innocent citizens and/or well-known public figures, and keeps this a secret

2. The power held by heads of state is second to that of small unknown groups who really control world politics

3. Secret organizations communicate with extraterrestrials, but keep this fact from the public

4. The spread of certain viruses and/or diseases is the result of the deliberate, concealed efforts of some organization

5. Groups of scientists manipulate, fabricate, or suppress evidence in order to deceive the public

6. The government permits or perpetrates acts of terrorism on its own soil, disguising its involvement

7. A small, secret group of people is responsible for making all major world decisions, such as going to war

8. Evidence of alien contact is being concealed from the public

9. Technology with mind-control capacities is used on people without their knowledge

10. New and advanced technology which would harm current industry is being suppressed

11. The government uses people as patsies to hide its involvement in criminal activity

12. Certain significant events have been the result of the activity of a small group who secretly manipulate world events

13. Some UFO sightings and rumors are planned or staged in order to distract the public from real alien contact

14. Experiments involving new drugs or technologies are routinely carried out on the public without their knowledge or consent

15. A lot of important information is deliberately concealed from the public out of self-interest
